# Association of perceived physician communication style with patient satisfaction, distress, cancer-related self-efficacy, and perceived control over the disease

**DOI:** 10.1038/sj.bjc.6600798

**Published:** 2003-03-04

**Authors:** R Zachariae, C G Pedersen, A B Jensen, E Ehrnrooth, P B Rossen, H von der Maase

**Affiliations:** 1 Psychooncology Research Unit, Aarhus University Hospital, Aarhus, Denmark; 2 Department of Oncology, Aarhus University Hospital, Aarhus, Denmark

**Keywords:** patient–physician relationship, communication skills, self-efficacy, anxiety, depression, anger

## Abstract

The aim of the study was to investigate the association of physician communication behaviours as perceived by the patient with patient reported satisfaction, distress, cancer-related self-efficacy, and perceived control over the disease in cancer patients. Questionnaires measuring distress, self-efficacy, and perceived control were completed prior to and after the consultation by 454 patients attending an oncology outpatient clinic. After the consultation, the patients also rated the physicians' communicative behaviours by completing a patient–physician relationship inventory (PPRI), and the physicians were asked to estimate patient satisfaction. The overall results showed that higher PPRI scores of physician attentiveness and empathy were associated with greater patient satisfaction, increased self-efficacy, and reduced emotional distress following the consultation. In contrast, lower PPRI scores were associated with reduced ability of the physician to estimate patient satisfaction. The results confirm and expand previous findings, suggesting that communication is a core clinical skill in oncology.

Patient dissatisfaction can lead to low understanding and recall of information, poor compliance, lengthier recovery periods, and increased complication rates (Fallowfield, 1992). Patients often do not recall and understand the information given, and when information is particularly upsetting, most patients are too stunned to register any further information given to them (Hogbin and Fallowfield, 1989). As a consequence, cancer patients often feel they lack information, which can lead to uncertainty, anxiety and depression (Newall *et al*, 1987). A review of the literature suggests that patient-centred approaches generally are associated with greater satisfaction, compliance, feelings of being understood, and resolution of patient concerns (Ong *et al*, 1995). Patient-centred interactions have been defined as those in which the patient's point of view is actively sought by the physician, which implies that the physician behaves in a manner that facilitates the patient to express himself, and that the patient feels free to speak openly and ask questions (Stewart, 1984). There is compelling evidence demonstrating that better physician–patient relationships are associated with improved health outcomes (Stewart, 1995; Ford *et al*, 1996), including greater symptom resolution, reduced stress, lower blood pressure in hypertensive patients (Orth *et al*, 1987), lower blood glucose levels in diabetics (Greenfield *et al*, 1988), and better postoperative pain control with reduced use of analgesics (Egbert *et al*, 1964).

It is primarily through communication that the relationship with the patient is forged (Gilligan and Raffin, 1997), and communication should thus be viewed as a core clinical skill (Fallowfield and Jenkins, 1999). It is an erroneous assumption that patients want technical expertise rather than good communication, this illogically implying an either/or situation (Fallowfield, 1992), and it has been shown that cancer patients are in fact generally willing to address their emotional and psychosocial functioning (Detmar *et al*, 2000). Physicians, on the other hand, are often inadequately trained in communication skills, which may lead to distancing and avoidance in discussing emotionally difficult communications with cancer patients (Baile *et al*, 1997). Although recognition of psychological distress is a crucial aspect of patient care, oncologists often fail to detect general distress in patients (Ford *et al*, 1994; Cull *et al*, 1995; Fallowfield *et al*, 2001) and ask few questions regarding patients' psychological health (Ford *et al*, 1996). Discrepancies found between physician-rated satisfaction and patient satisfaction indicate that physicians often perceive patients' affective responses inaccurately (Hall *et al*, 1999).

Research suggests that cancer patients who report greater efficacy with respect to their capacity to cope with the disease and its treatment are better adjusted and experience greater quality of life than patients who feel less efficacious (Merluzzi *et al*, 2001). Patients with high self-efficacy also have fewer episodes of negative psychological states, for example, depression, and tend to develop more realistic goals than patients with low self-efficacy (Bandura, 1997). An important aspect of disease-related self-efficacy is the sense of control and involvement in the treatment, and active involvement of patients in medical encounters has been associated with several desirable outcomes, including greater satisfaction, increased adherence to treatment, and positive treatment outcomes (Tennstedt, 2000). Disease-related self-efficacy may on the one hand be regarded as a predictor of how the patient perceives and copes with the encounter with the physician. On the other hand, self-efficacy may also be regarded as an outcome variable, that is, as a result of the physician–patient relationship. It will thus be of interest to investigate to what extent changes in patient self-efficacy following the patient–physician encounter are related to the patients' perceptions of physician communicative behaviours.

On this background, the aim of our study was to investigate the influence of patient perceived physician communication style on patient satisfaction, distress, self-efficacy, and perceived control over their disease. We also wished to explore the ability of the physicians to estimate patient satisfaction with the consultation. Results from previous studies led us to expect that increased communicative skills of the physicians as perceived by the patients would be associated with greater patient satisfaction (hypothesis 1), larger reductions in emotional distress (hypothesis 2), larger increases in cancer-related self-efficacy and perceived control over the disease (hypothesis 3), and greater ability of the physician to estimate patient satisfaction with the consultation (hypothesis 4).

There are several avenues of research into patient–physician communication. Some studies have used direct observations of a limited number of consultations (Hall *et al*, 1988) or structured patient interviews (Stewart, 1984; Butow *et al*, 1994), while others have used questionnaires (Cantwell and Ramirez, 1997; Detmar *et al*, 2000). To limit response and selection bias and to attain a clearer picture of the everyday physician–patient interactions in an oncology outpatient clinic, we chose to administer questionnaires to a large number of consecutively recruited patients before and after the consultation, while keeping both patients and physicians completely anonymous.

## PATIENTS, PHYSICIANS, AND METHODS

### Patients

All patients attending the outpatient clinic at the Department of Oncology, Aarhus University Hospital during a 4-week period were asked to participate. Prior to their decision, the patients were given brief verbal and written information about the aim and methods of the study. The recruitment was terminated, when a total of 500 patients had consented to participate.

### Physicians

All physicians at the department were likewise asked to participate in the study. At the time the staff consisted of 31 doctors, of whom 13 were specialists in oncology and 18 junior doctors in different training positions. Of the physicians there were 13 males and 18 females.

### Procedure

Participating patients received three closed envelopes with identical arbitrary codes. Envelope 1 was to be opened prior to the consultation and contained Patient-questionnaire 1. Envelope 2, which was to be opened after the consultation, contained Patient-questionnaire 2. Envelope 3 containing the Physician-questionnaire was to be handed over to the physician, who completed the questionnaire after the consultation. The codes enabled the pairing of patient and physician responses in the data analysis, while ensuring the anonymity of both.

### Questionnaires

#### Patient questionnaire 1

It consisted of (1) a Brief Mood Scale (BMS), originally consisting of 13 positive and negative moods. A factor analysis revealed three independent factors consisting of a total of nine descriptors: (1) Anxious mood (nervous, worried, scared, anxious), (2) depressed mood (sad, hopeless), and (3) angry mood (angry, furious, bitter). Each factor loading was higher than 0.60 and the difference between the highest and second highest factor loading was greater than higher than 0.30. Internal consistency coefficients (Cronbach's *α*) ranged from 0.90 (anxious mood) to 0.82 (depressed mood). The positive mood descriptors all loaded on more than one factor and were therefore omitted. A Total Distress score was calculated as the sum of all three moods. (2) A short 14-item version of the Cancer Behavior Inventory (CBI) (Merluzzi and Martinez Sanchez, 1997; Merluzzi *et al*, 2001), with the total score reflecting the patient's confidence in maintaining activity and independence, coping with treatment-related side effects, seeking social support, and maintaining a positive attitude. The scale had an internal consistency of 0.88. (3) A 4-item Perceived Control scale constructed to measure the patient's belief in his/her overall control over the cancer and the recurrence of cancer through his or her own thoughts or behaviours, and (4) Additional questions asked about age, sex, marital status, and educational background. The response format was 7-point Likert scales, and the internal consistency coefficient of the total scale was 0.86. The results are presented as percentage scores.

#### Patient questionnaire 2

It consisted of the BMS, the CBI, and the Perceived Control scale. Also included was a Physician–Patient Relationship Inventory (PPRI) (Pedersen *et al*, 2001; Zachariae *et al*, 2001). A factor analysis revealed two independent factors: (1) *Attentiveness and professional skills*, consisting of 10 items (e.g. ‘The physician wanted to understand, how I experienced things’ and ‘The physician gave me the opportunity to ask questions’) and (2) *Empathy*, consisting of four items (e.g. ‘The physician may have understood my words but not my feelings’). The reliability and preliminary validity of the PPRI had previously been tested in a group of women attending a mammography clinic, showing significant correlations with satisfaction with personal contact with the physician (*R*=0.66) and the handling of the medical aspects of the examination (*R*=0.30) and significant inverse correlations with reported embarrassment, pain, and discomfort during X-ray, ultrasound, and biopsy procedures (*R*=−0.21 to −0.52). The questionnaires had satisfactory internal consistencies ranging from 0.90 to 0.82. Finally, patients were asked to rate their satisfaction with (a) the personal contact with the physician, (b) the ability of the physician to handle the medical aspects of the patient's situation, (c) the perceived importance of (a), and (d) the perceived importance of (b). Owing to the limited time period between questionnaires 1 and 2, patients were explicitly instructed to respond as they felt ‘*now*’, irrespective of how they had felt prior to the consultation.

In the *Physician-questionnaire*, the physician was asked to indicate the type of referral and disease severity, rated as the aim of the current treatment (curative, life-prolonging, or palliative). The physicians were asked to rate the degree to which they focused on (a) biomedical aspects, (b) personal/subjective experiences, and (c) feelings during the consultation. They were also asked to rate their perception of patient satisfaction with (a) the personal contact with the physician, and (b) the ability of the physician to handle the medical aspects of the patient's situation, as well as their own satisfaction with (a) their ability to handle the medical aspects of the patient's situation and (b) their ability to establish personal contact.

### Ethics

The patients gave their informed written consent before entering the study, which had been approved by the local ethics committee.

### Statistics

Categorical data were analyzed with *χ*^2^-tests. Data regarded as continuous were analysed with independent or paired *t*-tests and analyses of variance (ANOVAs), with subsequent pairwise comparisons conducted with Scheffe *post hoc* tests, corrected for multiple comparisons. Correlation analyses were conducted by calculating Pearson's *R* for continuous data and Spearman's *ρ* for ordinal data. Additional analyses were conducted using multiple, stepwise and multiple, logistic regression analyses. A significance level of 0.05 (two-tailed) was chosen.

## RESULTS

### Patients

A total of 704 patients were approached before the number of consenting patients reached 500. The demographic data for the patients are shown in Table 1

**Table 1:** Patient demographics

	***N***	**%**	**% Men**	**Mean age**	**% Women**	**Mean age**
Patients asked to participate	704	100	—	—	—	—
Patients consenting to participate	500	71	—	—	—	—
Complete sets of questionnaires returned by patients and physicians	454^a^	65	34	49.3	66	56.1
						
*Referrals*						
Routine	179	40	32	40.9	68	56.8
Chemotherapy	111	25	36	60.2	64	56.4
Specific problems	84	19	22	48.5	78	54.6
Newly diagnosed	50	11	47	54.2	53	55.4
Radiotherapy	20	5	45	49.4	55	52.1
Total	444^b^	100	—	—	—	—
						
*Disease severity (aim of treatment)*						
Curative	265	62	33	43.0	67	55.1
Life prolonging	124	30	34	61.0	66	56.7
Palliative	33	8	39	58.9	61	60.7
Total	422^c^	100	—	—	—	—
						
*Reasons given for not wishing to participate*						
Unwilling to state specific reason	86	42	—	—	—	—
Unable to cope	54	26	—	—	—	—
Physical symptoms	16	8	—	—	—	—
Forgotten glasses	10	5	—	—	—	—
Other reasons	38	19	—	—	—	—
Total	204	100	—	—	—	—

a Fourteen patients had not reported their sex.

b Referral was not reported for 10 patients.

c Aim of treatment was not reported for 32 patients.

. Routine follow-up patients and patients in curative treatment were younger than the remaining patients (*P*<0.05). Significant differences were found between referral types and disease severity groups for both educational background and employment status (*P*<0.001). Patients with basic education were less likely to be in curative treatment than patients with medium and university education. Employed patients were more likely to be in curative treatment and pensioners more likely to be in life prolonging treatment. There were no differences in the proportion of men and women and no differences in marital status between either referral types or disease severity groups.

### Satisfaction

**Figure 1:**
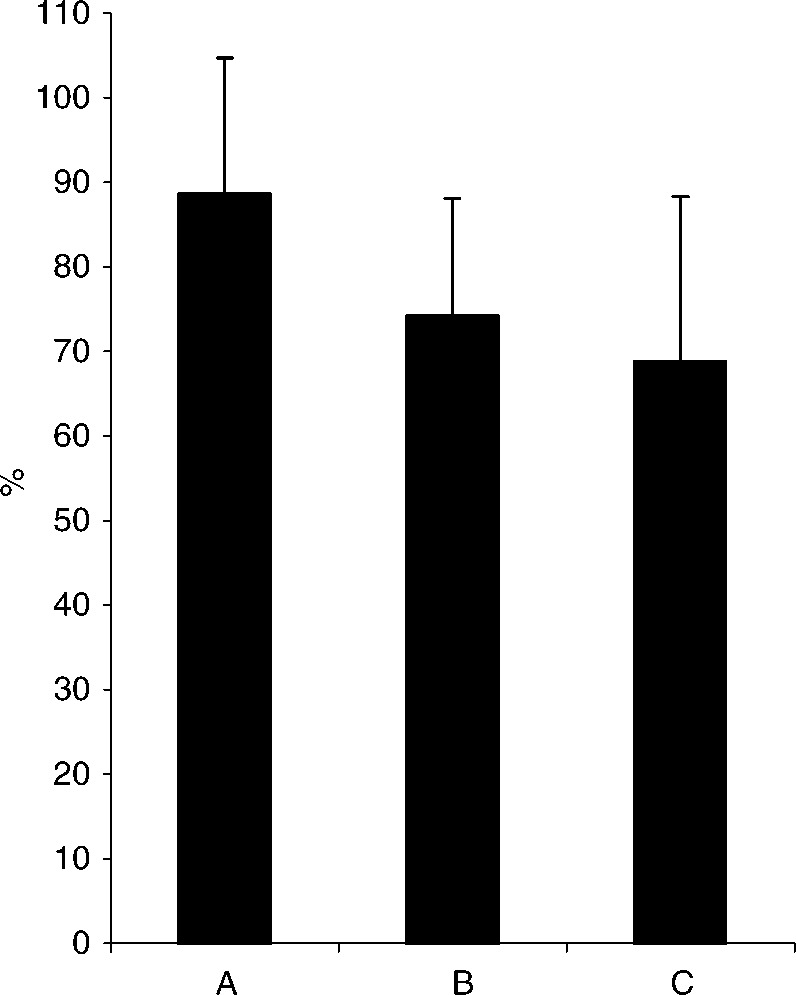
*Personal contact*: Mean percentage scores (s.d.) of patient satisfaction (**A**), the physicians' perception of patient satisfaction (**B**), and the physicians' satisfaction with his/her own ability to establish contact with the patient (**C**).

Mean patient satisfaction with personal contact with the physician (personal contact) and with the physician's handling of the medical aspects of the consultation (medical aspects) is shown in Figure 1

and 2

**Figure 2:**
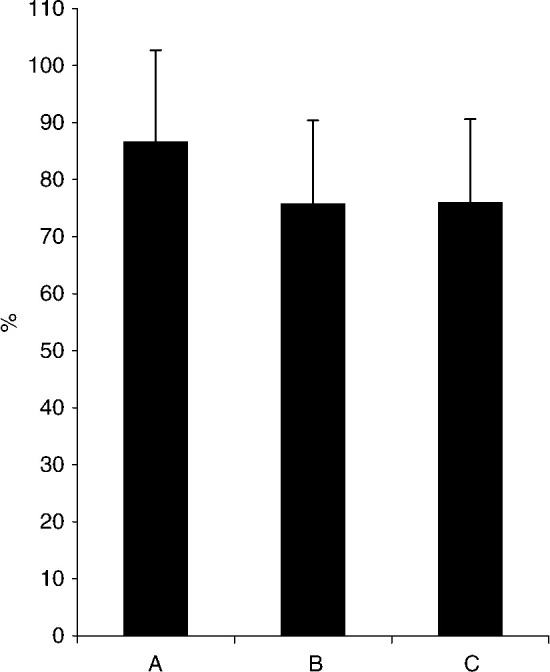
*Medical aspects*: Mean percentage scores (s.d.) of patient satisfaction (**A**), the physicians' perception of patient satisfaction (**B**), and the physicians' satisfaction with his/her own ability to handle the medical aspects of the patient's situation (**C**).

, together with physician ratings of patient satisfaction and physician satisfaction with his/her own ability to establish contact with the patient and handle the medical aspects. Patients in palliative care only were significantly (*P*<0.05) less satisfied than the two remaining severity groups. The physicians rated patient satisfaction with medical aspects slightly higher than patient satisfaction with personal contact (*P*<0.01), and were significantly (*P*<0.001) more satisfied with their ability to handle the medical aspects than with their ability to establish personal contact. In 81.5% of the consultations, the physicians were satisfied with the available amount of time. The physicians were less satisfied with the available amount of time for the consultation with patients in life-prolonging treatment (70.2%), compared to consultations with patients in curative (86.4%) and palliative treatment (84.4%) (*P*<0.001).

### Physician communication style

**Figure 3:**
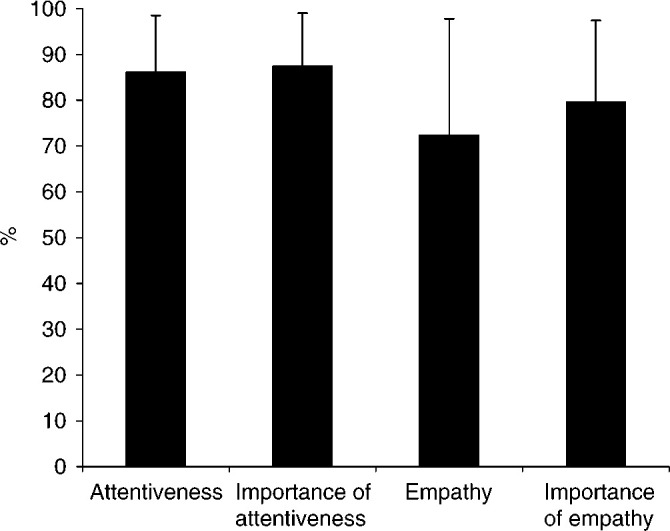
Mean percentage PPRI scores (s.d.) on the subscales of Attentiveness and Empathy and mean (s.d.) per cent rated importance of these skills.

The mean PPRI scores on the subscales of Attentiveness and Empathy and the mean per cent rated importance of these skills are shown in Figure 3

. When comparing the scores on the two subscales, patients rated physician Attentiveness significantly higher than Empathy, and the importance of Attentiveness higher than the importance of Empathy (*P*<0.001).

### Predictors of patient satisfaction (hypothesis 1)

As seen in Table 2

**Table 2:** Correlations between PPRI scores and outcome variables of patient satisfaction with personal contact and handling of medical aspects, Total Distress, cancer-related Self-efficacy, and Perceived Control over the disease

	**Satisfaction with personal contact**	**Satisfaction with handling of medical aspects**	**Change in Total Distress^a^^a^**	**Change in Self-efficacy**^a^	**Change in Perceived Control^a^**
PPRI Attentiveness	0.68**	0.64**	−0.13^a^	0.27**	−0.02
PPRI Empathy	0.38**	0.32**	−0.28**	0.18**	−0.03

a Partial correlations controlling for scores prior to the consultation.

** *P*<0.01

, significant (*P*<0.01) correlations were found between PPRI Attentiveness and Empathy scores and patient-rated satisfaction with personal contact and handling of medical aspects.

Patients scoring the Danish equivalents of ‘agree very much’, ‘agree’, and ‘in partial agreement’ were categorised as ‘satisfied’, while patients scoring ‘not sure’, ‘partially in disagreement’, ‘disagree’, and ‘disagree very much’ were categorised as dissatisfied. Using these categories, 11.1% of the patients were dissatisfied with the personal contact and 13.7% dissatisfied with the handling of the medical aspects. Multiple, logistic regression analyses were conducted with satisfaction/dissatisfaction as dependent variables, and with age, sex, marital status, educational background, disease severity, Self-efficacy, Perceived Control, moods (all measured prior to the consultation), and physician Attentiveness and Empathy entered as independent variables. Satisfaction with personal contact was associated with Perceived Control prior to the consultation (Odds Ratio (OR)=0.84, 95% CI=0.72–0.98; *P*<0.05), Attentiveness (OR=1.08, 95% CI= 1.01–1.16; *P*<0.05), and Empathy (OR=1.06, 95% CI=1.02–1.10; *P*<0.01). Thus, the less control over the disease and the greater physician Attentiveness and Empathy the patient experienced, the greater the likelihood that the patient would be satisfied with the personal contact with the physician. Greater satisfaction with medical aspects was associated with Attentiveness only (OR=1.12, 95% CI=1.05–1.18; *P*<0.001). No correlations were found between the physicians' rating of their own communication style (focus on biomedical, personal, and emotional aspects) and patient satisfaction scores (*r*=0.07–0.08).

Change scores of total Distress, Self-efficacy, and Perceived Control were calculated as the differences between scores prior to and after the consultation. Satisfaction with personal contact and medical aspects correlated moderately with change in Total Distress (*r*=−0.20; *P*<0.01) and change in Self-efficacy (*r*=0.15 and 0.18; *P*<0.01) but not with change in Perceived Control (*r*=0.02 and 0.03; NS). No correlations were found between the physicians' rating of their own communication style (focus on biomedical, personal, and emotional aspects) and change scores (*r*=−0.04 to 0.07).

### Predictors of changes in Distress, Self-efficacy, and Perceived Control (hypotheses 2 and 3)

As seen in Figure 4

**Figure 4:**
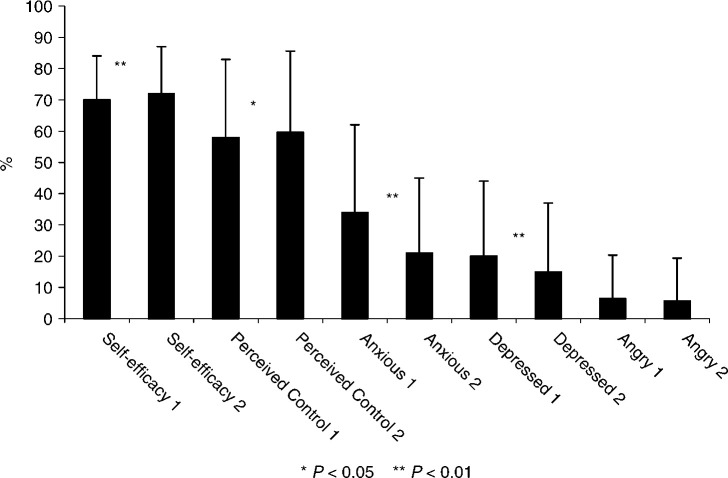
Mean percent scores (s.d.) of cancer-related self-efficacy, perceived control over disease, and mood prior to (1) and after the consultation (2).

, small, but significant, changes in Self-efficacy, Perceived Control, and moods were found after the consultation. Change scores of total Distress, Self-efficacy, and Perceived Control were calculated as the differences between pre- and postconsultation scores. No correlations were found between the physicians' ratings of their own communication style (focus on biomedical, personal, and emotional aspects) and change scores (*r* −0.04 to 0.07). When analysing change scores with MANOVA's, patients in palliative treatment were more anxious and angry after the consultation, while mean reductions in negative mood were found for the two remaining severity groups (*P*<0.05). No differences were found for sex, marital status, or education.

As seen in Table 2, significant (*P*<0.01) correlations were found between Attentiveness and Empathy scores and change scores of Distress, Self-efficacy, and Perceived Control, when controlling for scores prior to the consultation with partial correlations. To control for the possible influence of background variables, self-efficacy, and mood, a number of multiple, stepwise, linear regression analyses were conducted with change scores of total Distress, cancer-related Self-efficacy, and Perceived Control as dependent variables and age, sex, marital status, education (step 1), disease severity (step 2), Self-efficacy, Perceived Control (step 3), distress scores prior to the consultation (step 4), and physician Attentiveness and Empathy (step 5) entered as independent variables.

*Reduced total Distress* after the consultation was associated with living with a partner (*β*=−0.20; *R*^2^ change=0.04; *P* (F change)<0.01), low degree of Self-efficacy (*β*=0.11; *R*^2^ change=0.04; *P* (F change)<0.01), high distress prior to the consultation (*β*=−0.32; *R*^2^ change=0.08; *P* (F change)<0.001), and Empathy (*β*=−0.24; *R*^2^ change=0.06; *P* (F change)<0.01). *Increased cancer-related Self-efficacy* after the consultation was associated with low degree of Self-efficacy prior to the consultation (*β*=−0.42; *R*^2^ change=0.06; *P* (F change)<0.001), Perceived Control prior to the consultation (*β*=0.26; *R*^2^ change=0.05; *P* (F change)<0.002), and Attentiveness (*β*=0.30; *R*^2^ change=0.08; *P* (F change)<0.001). *Increased Perceived Control* over the disease after the consultation was associated with older age (*β*=0.24; *R*^2^ change=0.06; *P* (F change)<0.001) and lower degree of Perceived Control prior to the consultation (*β*=−0.23; *R*^2^ change=0.05; *P* (F change)<0.001).

### Physician ability to estimate patient satisfaction (hypothesis 4)

Significant (*P*<0.05), but small, correlations were found between patient satisfaction ratings and the corresponding physician estimates of patient satisfaction (*r*=0.12 and 0.13). Discrepancy scores were calculated as the difference between patient and physician ratings, with scores ranging from −100% to +100% with negative scores indicating that physicians had over-rated and positive scores indicating that physicians had under-rated patient satisfaction. Mean discrepancy scores were positive, indicating that physicians generally under-rated patient satisfaction (data not shown). Using the percentiles closest to 25 and 75%, consultations were then divided into three groups according to the discrepancy scores: (1) consultations where physicians had over-rated patient satisfaction (<25%), (2) moderate discrepancies (25<75%), and (3) consultations where physicians had underestimated patient satisfaction (>75%). Physicians over-rated satisfaction with personal contact of more patients in palliative treatment (21.4%), than patients in life-prolonging (5.6%) or curative treatment (8.5%) (*P*<0.05). Physicians also over-rated satisfaction with medical aspects of more patients in palliative (25.0%) than patients in life-prolonging (5.7%) or curative treatment (16.1%) (*P*<0.05). No associations were found with educational background. When analysing the association between PPRI scores and satisfaction discrepancies with ANOVAs, the results showed that in consultations, where physicians had over-rated patient satisfaction with personal contact, the physicians had been rated significantly lower on Attentiveness, than in the remaining consultations (*P*<0.001). In consultations where physicians had over-rated patient satisfaction with medical aspects, physicians were rated lower on both Attentiveness and Empathy (*P*<0.001). The results are shown in Table 3

**Table 3:** Mean patient ratings (±s.d.) of Attentiveness and Empathy for consultations where physicians have over-rated, where there was no discrepancy between patient and physician ratings, and where physicians had under-rated patient satisfaction with personal contact and medical aspects

	**Satisfaction with personal contact**		**Satisfaction with medical aspects**	
	**PPRI Attentiveness**	**PPRI Empathy**	**PPRI Attentiveness**	**PPRI Empathy**
Physician over-rated satisfaction	79.8±16.1***	69.4±22.7 ns	71.9±18.7***	60.3±21.9***
No discrepancy	88.2±9.7	73.4±25.7	88.1±9.2	75.4±24.0
Physician under-rated satisfaction	90.1±9.0	74.5±27.7	89.4±9.9	69.1±29.7

*** *P*<0.001 (Scheffe *post hoc* tests, corrected multiple comparisons). ns, not significant.

.

## DISCUSSION

The patients included in our study were generally satisfied with both the personal contact with the physician during the consultation and with the handling of the medical aspects of the situation. Only 11.1 and 13.7% of the patients were dissatisfied with the personal contact and handling of medical aspects of the consultation. This is considerably fewer than the median 38% dissatisfied hospital outpatients reported by Ley (1988), and could perhaps be a result of the cutoff used when dichotomising the data. The result, however, is comparable to the results of a recent survey of patient satisfaction at the Aarhus University Hospital oncology outpatient clinic (Service- og Kvalitetskontoret, 2000). Also, choosing a higher cutoff would have yielded more than 50% ‘dissatisfied’ patients; a number that does not correspond to the general experience at the clinic. Although we attempted to minimise possible bias by anonymising the respondents, our result could be an underestimation, as patients may have a tendency to give socially desirable responses because of anxiety that direct criticism of their physician could adversely affect their care (Fallowfield, 1992). Also, we do not know what the responses of the 204 patients who declined to participate would have been. Patients who are less healthy have been found to be less satisfied than healthier patients (Hall *et al*, 1998), a finding that is confirmed by our findings that patients in palliative care were less satisfied than the remaining patients. It should be noted that several factors, including expectations, aspirations, and perceived health status may influence and confound measurements of satisfaction of seriously ill patients (Avis *et al*, 1995; Fakhoury, 1998). Several patients gave physical exhaustion and/or psychological distress as their reason for not participating, and it is possible that inclusion of these patients would have led to reduced mean satisfaction. On the other hand, it is also possible that patients who were generally critical of their care would have made an extra effort to participate in the study. Although physicians were generally satisfied with the time available and their ability to establish contact and handle the medical aspects of the consultation, they were somewhat less satisfied than patients, and generally believed that the patients were less satisfied than they actually were, a finding which is consistent with results of other studies (Smith, 1986).

We found positive associations between patient satisfaction and the physicians' communicative behaviours as rated by the patients, even when controlling for sociodemographic factors, disease severity, self-efficacy, and distress prior to the consultation. The results thus confirmed *hypothesis 1* and are in support of previous results showing that higher frequency of patient-centred behaviours is associated with greater patient satisfaction (Stewart, 1984; Roter *et al*, 1987; Ong *et al*, 1995). That both Attentiveness and Empathy predicted satisfaction with personal contact suggests that both the physicians' behaviours with regard to listening, letting the patient ask questions, giving information, and explaining the biomedical aspects and their ability to respond to the patients' emotions are important to the patient–physician relationship. Satisfaction with the medical aspects was only associated with Attentiveness. This suggests that, although the two aspects are correlated, satisfaction with medical aspects and personal contact are two separate factors. Although the importance of attentiveness was rated slightly higher (7.4%) than the importance of empathy, our results also suggest that patients consider both aspects important, putting emphasis on the ability of the physicians to listen and communicate in a precise manner as well as their ability to respond to the emotional needs of the patients. The physicians' own ratings of their communicative style, for example, to what degree he/she focused on biomedical, personal, or emotional aspects, were uncorrelated with patient satisfaction. One reason could be that the physicians were unaware of several aspects of their behavior, as suggested by an observational study finding unrecognised behaviours in 16 out of 19 physicians (Smith, 1986).

As predicted by *hypothesis 2*, we found significant inverse relations between changes in total distress and patient ratings of both physician Attentiveness and Empathy. When controlling for initial distress levels as well as sociodemographic factors, disease severity, and self-efficacy, physician Empathy remained a significant predictor in the expected direction. Our results are in concordance with previous findings that a training course designed to enhance the physicians' emotion-handling skills was associated with reduced emotional distress in patients (Roter *et al*, 1995). We have no ready explanation as to why patients living with a partner reported greater reductions in distress than single, widowed, or divorced patients. It is possible that several of these patients had their partner present during the consultation, and that this factor had a positive influence on the distress levels of the patients. This, however, remains unclear, as we unfortunately did not ask, whether the patients had someone present during the consultation.

We also found positive correlations between both Attentiveness and Empathy and changes in cancer-related self-efficacy, confirming *hypothesis 3* that the aspects of the physicians communicative behaviour related to listening, explaining and letting the patient ask questions would be associated with increased disease-related self-efficacy. While the impact of specific interventions, that is, stimulation of patients' willingness to ask questions during the medical interview or general health education programmes, on patient self-efficacy, has been studied in several patient groups (Gifford *et al*, 1998; Tennstedt, 2000), we are not aware of any studies specifically investigating the association between physician communication style and self-efficacy of cancer patients. Cancer patients, who report greater efficacy with respect to their capacity to cope with the disease and its treatment, are better adjusted, experience greater quality of life, and are less distressed than patients who feel less efficacious (Bandura, 1997; Merluzzi *et al*, 2001), and further investigations of the importance of physician communication for self-efficacy of cancer patients are needed. Since cancer-related self-efficacy is primarily defined as the sense of control and involvement in the treatment and the active involvement of the patient in medical encounters, we also asked the patients about their perceived control over the disease and its possible recurrence. While perceived control correlated with self-efficacy, it was not associated with any aspects of physician communication style. Our results generally suggest that perceived control over the disease may be an independent aspect of illness perception, relatively unrelated to situational factors. This finding is in concordance with previous findings that even illusory positive beliefs of women with breast cancer about their disease and their ability to control disease were associated with positive mental health but independent of the objective medical evidence (Taylor *et al*, 1984).

Our results showed only very small correlations between patient satisfaction and the corresponding physician estimates of patient satisfaction. This confirms previous findings of substantial discrepancies between patient- and physician-rated satisfaction, indicating that patients' affective responses are often inaccurately perceived by the physicians (Hall *et al*, 1999). While the physicians generally under-rated patient satisfaction, they had over-rated satisfaction for a substantial number of consultations. When we analysed Attentiveness and Empathy scores of physicians for consultations, where the physicians had over-rated, under-rated, or correctly estimated patient satisfaction, the results showed significantly lower scores on patient-rated physician communication skills, when the physicians had over-rated patient satisfaction. Our results thus partly confirm *hypothesis 4* that greater communications skills would be associated with greater accuracy in the physicians' estimates of patient satisfaction. Reduced accuracy was, however, only the case, when physicians had over-rated patient satisfaction. One possible explanation could be a ceiling effect. When physicians under-rated satisfaction, patients were generally satisfied, which in turn would be associated with more adequate communication behaviours of the physicians.

Of special interest are our findings that patients in palliative care were more anxious and angry after the consultation and less satisfied with both their medical care and the personal contact with the physician than the remaining patients. The mean Attentiveness and Empathy scores of this group did not differ significantly from the scores of the remaining patients, which could suggest that the patient category needing it most was not receiving adequate Attentiveness and Empathy, an interpretation supported by our finding that physicians tended to over-rate the satisfaction of these patients. Since patients who are less healthy have been found to be less satisfied than healthier patients (Hall *et al*, 1998), it is also possible that the greater dissatisfaction of patients in palliative care only is primarily related to the severity of their disease. Further research is clearly needed.

A number of limitations to our study, because of our choice of methodology, should be noted. First, we chose not to use a direct measure of physician communication, but a measure of the patients' perception of their communicative behaviour. While direct observations of physician–patient interactions by neutral observers may provide a more objective measure of physician communication, such research procedures are, because of lack of anonymity, more likely to influence physician and patient behaviours and to introduce response bias. The results may therefore not be representative of patient–physician interactions in the busy day-to-day practice in an oncology outpatient clinic. To limit the possibility of selection and response bias, we chose to keep both patients and physicians completely anonymous. We thus refrained from asking about the gender and the training of the individual physician. Unfortunately, we did not ask the physician to record whether the patients had a partner with them during the consultation. We are therefore unable to assess the possible influence of these factors. Although our response rate was acceptable, approximately 30% of the patients declined to participate, and we do not know to what degree these patients differ from the participants. Since questions concerning patient satisfaction and physician behaviour could be biased because of their susceptibility to social desirability, the validity of further studies could benefit from the inclusion of a measure of social desirability, for example, the Marlow–Crowne Social Desirability Scale (Marlowe and Crowne, 1977). Finally, one should be cautious in the interpretation of cause and effect, for example, of the influence of physician communication on patient self-efficacy. Although we used a pre–post design, testing specific hypotheses, our data are still of a correlational nature, and the associations found do not necessarily indicate a causal relation. Only truly experimental designs, for example, randomising physicians to communication training or a control group, offer this possibility.

In conclusion, our results generally confirmed our hypotheses that the patient perceived physician communication skills would be associated with both patient satisfaction and changes in patient distress and self-efficacy following the consultations. Physicians were often inaccurate in their estimations of patient satisfaction, and the results partially confirmed our hypothesis that accuracy was associated with physician communication skills.
